# Does a Pandemic Preempt Participatory Medicine?

**DOI:** 10.2196/23860

**Published:** 2020-12-14

**Authors:** Michael Louis Millenson

**Affiliations:** 1 Health Quality Advisors LLC United States; 2 Feinberg School of Medicine Northwestern University Evanston, IL United States

**Keywords:** participatory medicine, COVID-19, pandemic, Fitbit, DETECT study, Body Politic, wearables, sensors, patient-generated health data, shared decision making

## Abstract

For those of us who believe deeply in a collaborative relationship between patients and doctors, the chaos created by the COVID-19 pandemic has brought an uncomfortable question to the fore: Is participatory medicine still relevant during a pandemic? Drawing liberally upon the Jewish tradition of Talmudic reasoning, I would like to offer 3 considered replies: “Yes,” “no,” and “it depends.” Sometimes, patients may have no choice but to cede control to medical professionals, even though patients are still the experts on their own lives. Other times, the shared control of participatory medicine is both an ethical and clinical imperative. However, as the worldwide toll exacted by COVID-19 has made us grimly aware, no one is really in control. That is why, in these uncertain times, the path forward requires maintaining mutual trust between health care providers and patients, whatever the circumstances. After all, it is our bodies and our selves at stake.

Consider the following scenario: you are at home with your significant other, and that low-grade fever you have been running is rising. You have also developed a persistent cough, and you are losing your sense of smell. In a panic, you phone your doctor, who tells you to go immediately to a special area set aside for patients with COVID-19 by your local hospital. He will call ahead.

“Thank you so much, doctor,” you blurt out. Yet even in your dazed state, you know there are a few more questions you just have to ask.

“As an informed consumer,” you begin, “I’m wondering if the hospital’s online price list includes COVID-19 treatment costs.” You add, “I also want to make sure the emergency room physicians understand that I expect to be a partner in the coproduction of my care [1]—you know, full access to my electronic health record, including clinical notes. I prefer an API (application programming interface) readable format [2], but I’m willing to accept a PDF emailed to me every evening.”

Your significant other violently grabs the phone and starts apologizing profusely. “I’m really sorry,” they tell the doctor. “That must be the fever talking. We’re on our way to the hospital right now.”

For those of us who believe deeply in a collaborative relationship between patients and doctors, the chaos created by the COVID-19 pandemic has brought an uncomfortable question to the fore: Is participatory medicine still relevant during a pandemic? Drawing liberally upon the Jewish tradition of Talmudic reasoning, I would like to offer 3 considered replies: “Yes,” “no,” and “it depends.”

Let us start by expanding on the “no” answer. The term “participatory medicine” [3] contains 2 important assumptions. The first is that the patient and the patient’s family (ie, a term used here to include all those the patient chooses to involve in care) are in a position to participate. The second assumption is that this participation adds information of value.

Neither of these assumptions is invariably true. Someone rushed into surgery after a serious accident, for example, is in no position to speak up with a perspective on how the surgeon should close their wounds, nor would it be helpful to engage in shared decision making about staples versus sutures. A drug allergy or similar critical information that a family member or caregiver might provide is, of course, a different matter. Unfortunately, caring for a SARS-CoV-2 infection still too often feels like an emergency. There is extraordinary stress not just on patients and family, but also on the medical staff, as they, too, worry about getting sick or infecting others [4]. Perhaps most importantly, there is tremendous clinical uncertainty. Participatory medicine may sometimes have to take a temporary back seat to avoid being a harmful distraction. However, there are other circumstances in which participatory medicine could be relevant and constitute the cornerstone of an effective COVID-19 response.

To be clear, the term “participatory” in this context does not mean the kind of public health actions that everyone, infected or not, should be participating in (eg, wearing a mask, practicing social distancing, and washing one’s hands). Rather, it refers to the deliberate incorporation of the patient voice into the national COVID-19 response. For instance, in June 2020, the National Institutes of Health launched a data analytics platform, and its usefulness will ultimately depend upon Americans with COVID-19 sharing their electronic health record information. This platform was opened to researchers in September 2020, and it incorporates analytic techniques that are designed to examine everything from potential COVID-19 risk factors to the effectiveness of different therapies [5].

The pandemic has prompted clinicians, employers, and public health officials to ask individuals to self-monitor at different times, such as when they are healthy, if and when they become symptomatic, and during recuperation. Digital tools for web-based symptom checks and consultations are key components of combating the COVID-19 pandemic [6]. As a result, we may be entering a new age of prioritizing digitized, patient-generated health data. This is in essence the mainstreaming of the “e-patient,” a term coined by Dr Tom Ferguson to describe individuals who are “equipped, enabled, empowered and engaged in their health and health care decisions” [7]. 

To give an example of the central role being played by patient-generated health data, Fitbit Inc has developed a COVID-19 symptom tracking service called Ready for Work. It lets employees self-report their symptoms on a Fitbit device and lets employers see worker information on a central dashboard. This helps employees and employers determine when it is safe to return to the workplace. Preliminary data from a nonpeer-reviewed Fitbit study written in mid-August 2020 has suggested that “hospitalization risk can be calculated from self-reported symptoms, and relevant and predictive physiological signs related to COVID-19 may be detected by consumer-wearable devices” [8].

Similar initial results were reported at the end of October 2020 by the Scripps Research Translational Institute in its DETECT (Digital Engagement and Tracking for Early Control and Treatment) study, which involved large-scale epidemiological research on consumers who use a wide range of smart wearables [9]. Additionally, in one estimate, the number of sales for wearables capable of tracking and monitoring COVID-19 symptoms and other conditions will jump from 30 million devices in 2020 to 104 million by 2025 [10].

Clinical uncertainty about COVID-19 has also given birth to online patient support groups, such as Survivor Corps, COVID-19 Recovered - Survivors, and the Body Politic COVID-19 support group. Participation in these support groups now includes tens of thousands of individuals around the globe. As Fiona Lowenstein, founder of one such group, wrote in Vox, “As we wait for institutions to catch up with a new and fast-moving virus, parallel forms of information-sharing via communities, personal stories, and support groups…have become crucial” [11].

Now that we have seen the “yes” and “no” arguments for the relevance of pandemic-era participatory medicine, here is the last answer: “It depends.”

Listen for a moment to a woman named Byllye Avery recounting what she learned from fellow patients after a traumatic encounter with the health care system. “If you don’t know how to take care of yourself, you are basically ignorant,” she wrote. “And health information,” she added, “had to be shared within the context of one’s life. [There was a] right to have medical information and…patient participation.”

That advice was given to Avery nearly a half century ago at a 1971 women's health meeting whose attendees were part of a group that authored the book, *"Our Bodies, Ourselves".* Avery, who later founded the Black Women’s Health Imperative, shared her memories in a preface to that groundbreaking book’s 25th anniversary edition [12]. As noted in a history of participatory medicine, the movement owes a large debt to feminists [13].

Although it has taken decades, the health care system has finally accepted the principles of participatory medicine as valid. However, putting all these principles into practice may not always be possible, especially at a time when patients, their friends and loved ones, and health care providers are all extraordinarily stressed by the mortal threat of a dangerous and incompletely understood pandemic. Health care, like much of life, is complicated and messy. Evolving circumstances dictate the appropriate response. Nonetheless, whether during a period of pandemic or relative placidity, certain bedrock principles must remain part of the relationship between professionals and patients. These principles include honesty, mutual respect, and the mutual sharing of information. Such sharing needs to continue, even when it means admitting to uncertainty, fear, guilt, or other uncomfortable emotions.

Sometimes, patients may have no choice but to cede control to medical professionals. This decision, though, does not relieve medical professionals of the obligation to listen to patient and family concerns; patients are still the experts on their own lives. Other times, the shared control of participatory medicine is both an ethical and clinical imperative [14]. However, as the worldwide toll exacted by COVID-19 has made us grimly aware, no one is really in control. That is why, in these uncertain times, the path forward requires maintaining mutual trust between health care providers and patients, whatever the circumstances. After all, it is our bodies and our selves at stake.

## Abbreviations

API: application programming interface
DETECT: Digital Engagement and Tracking for Early Control and Treatment
